# Patients’ voices from music therapy at a South African psychiatric hospital

**DOI:** 10.4102/sajpsychiatry.v28i0.1884

**Published:** 2022-07-29

**Authors:** Carol Lotter, Werdie van Staden

**Affiliations:** 1 Department of Music, Faculty of Humanities, University of Pretoria, Pretoria, South Africa; 2 Centre for Ethics and Philosophy of Health Sciences, Faculty of Health Sciences, University of Pretoria, Pretoria, South Africa

**Keywords:** music therapy, verbal expression, musical participation, patient voices, adult mental health

## Abstract

**Background:**

In the Life Esidimeni tragedy, crucial voices of mental healthcare users and practitioners were silenced, captured in the Ombud’s report as a ‘failure to listen’. Working against this kind of failure, various therapeutic interventions listen deliberately and uncover the voice of the patient, that is, what matters from his or her subjective perspective in his or her particular circumstances. Amongst these interventions, music therapy provides for this sensitive listening by expanding the scope and means of expression from the verbal to the musical.

**Aim:**

This article reports on a qualitative exploration of patients’ lived experiences both during and after their course of individual music therapy, expressed both verbally and in the language of active music-making.

**Setting:**

A tertiary public psychiatric hospital in South Africa.

**Methods:**

Audio-video recordings of 131 music therapy sessions and 15 post-therapy interviews were analysed thematically. From three sets of themes accounting for patients’ verbal contents, musical participation and verbal post-therapy reflections, 11 salient voices were identified.

**Results:**

The 11 voices that emerged were (1) the voice of struggle, (2) the voice of disturbance, (3) the voice that feels, (4) the voice of isolation, (5) the powerless voice, (6) the voice that desires, (7) the voice of flow and connection, (8) the reflecting voice, (9) the symbolic voice, (10) the resilient voice and (11) the voice of liberation.

**Conclusion:**

Although mental health practitioners may recognise these voices from their clinical experience, space and opportunity for hearing the voice of each patient should be generated deliberately.

## Introduction

South Africa experienced the deaths of more than 140 psychiatric patients in the Life Esidimeni tragedy amidst dehumanising circumstances that had been invoked by the Gauteng provincial government, in which about 1400 patients were hastily transferred during 2016 to mostly inadequate facilities.^1^ Within this tragedy was the deafening silence of the voice of patients. If they did speak, no one seemingly listened or what they had to say did not truly matter enough in averting their deaths.^1^ Ironically, it is ‘hearing voices’ that constitutes a common problem in the care and treatment of patients with mental illnesses. As part of standard care, these ‘voices’ are treated with the effect of silencing their incapacitating impact. In the tragedy, however, important voices of patients and practitioners were silenced. A chapter heading of the Ombud’s report on the tragedy poignantly captures this as a ‘failure to listen’.^1^

Another silencing may result from mental illness amongst patients for whom the combined impact of dire life circumstances and chronic mental illness is overwhelming.^2^ A mental illness and its diagnosis often come with stigma, isolation and loss, in some instances reducing the individual to nothing but a ‘mental patient’.^2,3^ The individual is seen through the lens of clinical features, a diagnostic label or even just a patient number, delimiting the narrative of the patient as ill, abnormal, weak, pathological and disabled. This narrative may render the patient powerless, voiceless and without agency.^2,3^

There are multiple complexities operating in silencing the voice of patients. These underscore the need for accounting in clinical and scholarly ways for their voice with even more sensitivity when compromised by mental illness, stigmatisation and the professionally legitimate filters of diagnostic and other service delivery systems. Clinicians in standard clinical mental healthcare engage more or less with the patient’s voice, that is, that which matters to the patient from his or her subjective perspective in his or her particular circumstances. More formalised therapeutic interventions of various kinds deliberately seek out and interact therapeutically with the subjective voice of the patient. One kind of intervention that does this is music therapy, for which music therapists in South Africa need to be duly registered at the Health Professions Council of South Africa.

Like other therapies, music therapy deliberately creates a space for the patient’s voice through the music therapist’s sensitive and empathic listening. Music therapy, however, expands the scope and means of expression from the verbal to the musical.^3^ Music therapy does not require formal musical education from the patient but mobilises musical expression that is suited to the patient’s abilities. These are usually the ordinary and intuitive abilities of listening to music, singing, drumming, making musical expressions on percussive instruments and improvisation on tonal instruments. Patients in music therapy express their voices within an interpersonal space with the music therapist in which thoughts, emotions and patterns of interaction unfold both in verbal and musical language.

Scholarly accounts of this listening in music therapy were derived from a study conducted in South Africa at the time of the Life Esidimeni tragedy. This study was conducted amongst inpatients at a large psychiatric hospital who were diagnosed with either a major depressive disorder (MDD) or a schizophrenia spectrum or other psychotic disorder (SSD). The interests of this study were on the qualitative verbal and musical affordances of using various music therapy methods during individual therapy and patient reflections after the course of music therapy.^4^ The verbal affordances within music therapy sessions were reported elsewhere,^5^ which showed responses within a therapeutic relationship that expressed interpersonal and intrapersonal connection, increased motivation, grappling with difficulty, emotional expression and the reclamation of energy, spontaneity and resilience.

Reflections after the course of music therapy were also reported elsewhere.^6^ These comprised participants’ praise for music therapy, the distress from which change emerged and various perceived gains cast in the wake of their distress. The perceived gains were new perspectives, growing strong, emotional fulfilment, becoming socially closer and more adept and becoming liberated and creatively inspired. These patient perspectives on a completed course of music therapy augment the evidence base established in clinician terms of what matters as a potential gain from music therapy for a MDD or SSD.

Notwithstanding the foci of these previous reports, the aim of this article was broader and distinctly foregrounded by the Life Esidimeni tragedy, albeit not about it. The aim was to explore voices of patients at a higher and more integrated level than in the previous reports, thereby accounting qualitatively for their lived experiences both during and after their course of individual music therapy, expressed both verbally and in the language of active music-making.

## Method

### Design

This study followed a qualitative case study design adopting an interpretivist epistemology.^7,8^ Although expressed in an integrated way during music therapy,^9^ two kinds of qualitative data were explored: firstly, verbal expressions of each patient during music therapy sessions arising in the conversations between the patient and the therapist, including when listening to music and during active music-making, as well as the patients’ verbal reflections after completing the course of music therapy. Secondly, qualitative data were the musical qualities during active music-making, which included the tone pitch, timbre, dynamics (tone volume and its changing), phrasing, melody, harmony, metre, rhythm, instrumentation and other conventional musical descriptors as applied to the conjoint music-making of patient and therapist.

### Participants

Participants had been purposively sampled before the commencement of a structured course of music therapy during their hospitalisation for either a MDD or a schizophrenia-spectrum or other psychotic disorder (SSD). There were 20 participants with these clinical diagnoses without verification through research criteria, 10 per diagnostic group, eight male and 12 female, with ages ranging between 18 and 57 years. They participated in a total of 131 individual music therapy sessions and 15 reflective interviews after the course of therapy. The remaining five participants had been discharged from hospital before reflective interviews could be performed.

### Procedures and data collection

Data were collected over a two-year period at a public psychiatric hospital in South Africa. Participants, after granting written informed consent, underwent individual music therapy structured in time-limited fashion, that being twice weekly for a total of eight sessions congruent with local resource constraints and the usual duration of hospitalisation. Sessions were structured uniformly across participants specifically for each session, but these flexibly accommodated the nuances of each patient’s unique process. The structuring provided for active and receptive music therapy methods in a blended fashion.^10,11^ These were (1) guided relaxation and music listening, (2) structured drumming exercises, (3) unstructured improvisations that were developed and extended during each session, (4) movement exercises, (5) vocal work, focusing on both structured songs and free improvisation, (6) a range of music listening methods comprising a range of arts therapies modalities and (7) song-writing.

The sessions were audio-video recorded for the purposes of transcription of verbal materials, description of music-making qualities and in-depth analyses. Patients’ participation in active music-making processes was described in detail by the therapist-researcher (C.L.) after each session using the audio-visual recordings. A deliberate continuous effort was made not to infer clinical interpretations from the patient’s music-making at the stage of data collection other than describing as unequivocally as possible the qualities of music making (e.g. loud, tentative, fast) and movement (e.g. stiff, flexible, energetic). These descriptions were verified by an independent music therapist.

At the conclusion of a course of music therapy, 14 patients who had completed eight sessions and one patient who had completed seven sessions were each interviewed by one of us (C.L.) using a semistructured interview guide (available upon request) that had been pre-approved both by a scientific advisory and a research ethics committee.

### Data processing and analysis

All verbal contents were transcribed verbatim. These verbal transcriptions and the detailed descriptions of the music-making qualities of each session resulted in three sets of qualitative data, comprising patient responses in (1) musical participation, (2) in-therapy verbal participation and (3) post-therapy verbal reflections, respectively.

These three data sets were first analysed separately, guided by grounded theory and thematic analyses.^12,13^ Data were segmented into first-level codes using open coding and then analysed inductively by constant and systematic comparisons, seeking emerging concepts that capture stable patterns. Higher-level codes so derived were subjected to the same inductive process until no new properties emerged from the continued coding and theoretical saturation was thus reached. The analyses were steered by the principle that the higher-order codes, subthemes and themes should remain an apt expression of the raw data, averting or ‘bracketing’ the imposition of prior theory as far as possible.^14,15^ The given content was honoured, capturing the ‘whatness’ of the very experience as distinct from the researchers’ interpretation of the experience,^16^ thus pursuing a description true to the voice of the participants. For this reason, subthemes and themes were identified without consideration of diagnostic categories. Guided by grounded theory, analyses were undertaken as a conjoint activity of the authors, revisiting and revising prior articulations iteratively. To ensure that the analysis of a large amount of data is credible and transparent, the coding process was systematically and comprehensively annotated using conceptual memoing through cycles of coding.^12^

For this article specifically, the preceding analyses were extended in the same way by comparing and organising subthemes across the three sets of data into higher-order codes as a means of relating the themes to each other.^17^ These higher-order codes were articulated as ‘voices’ in remaining true to the qualitative intent of this article, which was to place the personal responses of the patient at the centre of the analysis notwithstanding its integrative and high-level analytic aim.

### Ethical considerations

Ethical clearance to conduct this study was obtained from the Faculty of Health Sciences Research Ethics Committee at the University of Pretoria (reference number: 434/2013). Permission to conduct the study was obtained from the Chief Executive Officer of the hospital.

## Results

Patients are heard here in 11 salient voices, which represent the dominant descriptions of patients’ lived experiences during music therapy sessions and their post-therapy reflections. For each voice, we provide quotes that capture the particular voice most aptly, indicating the participant number, gender and session number or post interview as reference.

### The voice of struggle

‘…sadness and hazardous. It’s sadness that pushes you to the edge and you want to escape to somewhere where you will feel at peace, or end it all. … There was a time, *ja*, when I wish I was, *ja* [*shakes her head*]. Makes me feel hopeless, just on my knees. I can’t stand up. I’m trying to stand up. Maybe I’ll stand up when I get out of here … because there’s been 15 years, sometimes it makes me so hopeless that in these 15 years, why can’t I …? It’s a long pattern, and it’s a long time of living with this.’ (Participant A, female, session 01)

Not being able to feel, feelings of indifference, suppressing unbearable feelings and being afraid of feeling and facing emotions were all articulations of the voice of struggle. Struggling was also voiced through references to suicidal ideation, doubts and scepticism about facing a dreaded future and experiencing an impasse. Reality is deemed as hardship and struggle and described as ‘the worst journey to be endured’. This voice indicated a desire to escape an unbearable reality to an idealised situation. The disabling impact of trauma, fear of ongoing hurt, pain and fearing an unknown future perceived as ominous are all expressions of struggle. Furthermore, the struggle is embodied through indications of bodily distress, tiredness and feelings of stress and tension.

### The voice of disturbance

‘I am getting confused. I can’t think too much.’ (Participant T, male, session 01)

‘I’m stuck. I feel blocked. I don’t know.’ (Participant P, female, session 07)

Taken from the therapist-researcher’s description of video footage:

‘[*T*]he client plays a few beats on the drum and then stops. He copies the rhythm in a delayed manner. It is as if the client is playing in slow motion and struggling to focus. His playing becomes fragmented.’ (Participant T, male, session 02)

The voice of disturbance was expressed both verbally and musically. When reflecting on the music therapy process in the post-therapy interview, patients reported being in an impaired state prior to music therapy and experiencing improvisation as disturbing and discomforting as well as having difficulty with concentrating, being present in the here and now and remembering. In musical participation, patients expressed feelings of being stuck or blocked, a lack of self-confidence that limited accessing and expressing new musical ideas. Irregularity, interrupted flow and limited variation and range of musical expression were present in music-making. A disturbance was also evident in an incongruence between affect and musical expression, as well as rigidity and perseveration in musical expression. Low energy, blunted affect, lethargy, tremors and stiffness suggested disturbance expressed in bodily functions.

### The voice that feels

‘To tell you the truth, I’m so scared of being hurt; at this stage, I’m vulnerable.’ (Participant A, female, session 03)

‘I touched some feelings that I didn’t want to touch, but yet by touching them, I felt the feelings in a different way: it opened up feelings and memories that, um, the moment that you connect feelings to memories, which I haven’t done in a very long time.’ (Participant G, male, post interview)

Although emotional expression may seem an obvious result from this therapeutic context, the voice of feeling is underscored as it meaningfully captured a deepening of emotional expression and reference to specific internal states. One patient, when reflecting on an image she had drawn had this to say: ‘sadness, disappointment, regret … just some of the emotions I keep, that I bottle up, so I want it to be out in the open’ (Participant I, female, session 07).

Through verbal responses, patients expressed feelings of sadness, being heartbroken, feeling smashed and broken and feelings of hopelessness and experiencing themselves as useless. Pervasive feelings included vulnerability, anger towards themselves and others and difficulty trusting others. Weeping commonly emerged during active music-making and intensified emotional expression was sounded through musical dynamics and sentiments expressed in the qualities of musical expression.

### The voice of isolation

‘I feel scared and lonely. … I feel like there is no one, that it’s only me in the world. … I’m scared to be around people, sometimes.’ (Participant E, female, session 07)

Through musical participation, the voice of isolation was expressed through limited ‘in the moment’ awareness and difficulty engaging within the therapeutic musical relationship, as evidenced by the vacant stare, averted gaze, blunted affect and low levels of self-confidence, all preventing patients from venturing towards interactive musical experiences. Through verbal expression, this voice lamented loneliness, isolation and being away from family and social networks. The isolation was connected to rejection, loss, ‘being alone with my problems’ and neglect.

### The powerless voice

‘I’m very, very angry, I’m feeling very angry, *ja*, because I’m, I expected a place of, um, where at least basic things are there. … I feel so disappointed. When I found the basic needs are not [met], I ask myself what kind of a world, what kind of a place are we living in where people can just do things as they wish? … What I’m going through? I’m so angry and helpless because I can’t go and report this … so it puts me into a corner where I can’t say this one is wrong … I’m far from home, so I’m caught between here and here.’ (Participant A, female, session 06)

Powerlessness was expressed through references to being emotionally stuck, not being able to change circumstances, being at the mercy of the decisions of others, limited opportunities because of economic and education status, an impasse in being dependent on others, not being able to change one’s response to the debilitating effects of trauma, perceiving oneself as weak or useless and limited belief in one’s own capacity to be creative or contribute to musical participation.

### The voice that desires

‘I need someone. *Ja*, someone to share my feelings – my inner feelings. I want to be loved; I need that.’ (Participant F, male, session 07)

‘But there are personal things … having a boyfriend, having a husband, having children. I feel like I have been robbed of all of that.’ (Participant A, female, session 03)

This voice articulated the desire for romantic love and social connection, financial independence, to be with family, the restoration of family relations, to access one’s inner voice, freedom, growth, happiness and to live with increased energy.

### The voice of flow and connection

Taken from a description of video footage:

‘[*T*]he patient started playing a “laid-back” rhythm at a moderate tempo, playing the conga drums almost as if he had a pair of brushes with him. I supported his music on the piano, playing a jazz or swing improvised melody and chord sequence … the feeling in the joint music was comfortable. The patient played in a varied and creative manner, engaging by taking initiative in the rhythm and varying the timbre on the woodblocks. He was responsive to my imitations, and spontaneously reciprocated a glissando in ending the improvisation, upon which we both laughed.’ (Participant F, male, session 08)

The voice of flow and connection contrasts with the earlier-described *voice of disturbance*, where features of interrupted flow, fragmentation and disorganisation were observed in musical and verbal expression. In active music-making, flow and connection were experienced through regularity, flow, stability, expressive and natural movement, increased engagement, reciprocal responding and experiences of holding and resolution through supportive, interactive musical expression. A longing for personal connection was expressed verbally, as was the realisation of this connection in the sessions, where it was appraised as valuable to be sharing personally within the music space, being together and growing in interaction.

### The reflecting voice

‘I kind of got too close with myself, I got in touch with myself, my inner self … it made me come back to life.’ (Participant A, female, post interview)

‘[I]t opened my mind a bit. Life after being … death, because the main reason I am here is because I was thinking about dying, it gives me some reason not to commit suicide or to think about dying … life goes on.’ (Participant F, male, post interview)

Reflections on how music therapy was experienced included spontaneous commentary on active music-making, making choices in music-making, reporting on never having made music before and reflecting on reconnecting with music through personal associations. In the post-therapy interviews, patients reflected on music focusing the mind, being in touch with emotions, being exposed to new experiences and gaining new self-realisations. This voice shared how perspectives were gained and changed, how connections were made between past, present and future and how an awareness of spirituality was renewed. Patients reflected on how music elicited memories and how music opened up and facilitated dealing with old wounds. One patient captured this sentiment amongst participants as ‘wanted feelings in and unwanted feelings out’. Reflections also concerned the impact of music, music as an essence of being and the uniqueness of experiencing music therapy and aspects of sessions that were experienced as frustrating and not enjoyable.

### The symbolic voice

This voice spoke metaphorically using images, associations, symbols, memories, feeling states and musical experiences, through which patients explored and shared their stories. Metaphors ranged from concrete associations with people and objects to images and symbols of an abstract nature. The following example illustrates how the symbolic voice was activated during music listening, active musical participation and verbal reflection. One patient formed a clay image, representing a barrier line, depicting herself standing alone under a dark cloud and separated from her family, who were placed on the other side of the barrier. The patient then used the symbol of the barrier to talk about her lived experience of depression: ‘I feel like there’s a barrier between my family and I’m under a dark cloud alone. … I think my depression is my barrier line. That’s why I’m here.’ (Participant I, female, session 04).

A second piece of music invited the patient to imagine the clay scenario transformed in some way, however small the envisaged change may be. The patient reworked the clay image to represent the sun, her family reunited and a face depicting a smile, saying:

‘[*T*]he barrier makes me stronger. I can start dealing with it. If I go there, if I work through it, it can make me stronger. … Break the barrier. Keep on breaking the barrier. Even if it’s just … at a time.’ (Participant I, female, session 04)

Then the patient reflected on the symbol of the barrier through active music-making, by leading an improvisation on the conga drum supported by the therapist on the piano, which she titled ‘Decimal breaking the barrier’. As she played, she provided a verbal account of the unfolding musical narrative, concluding by saying:

‘…not expecting too much of myself – so I can’t expect to break all of the barrier in a short time – I must give myself time’. (Participant I, female, session 04)

This example demonstrates how the combined creative act of music listening, image-making, verbal reflection and music-making offers the voice of symbolism to process and integrate aspects of patients’ lived experience.

### The resilient voice

‘…that I’ll be stronger than the pain, that I’m going to fight it. To be resilient. Even though I’ll fall, I will stand up and move on. No more being scared of taking chances. And even if that means taking the first step, saying I’m no longer scared.’ (Participant A, female, session 08)

‘Stronger than the pain’ aptly captured this voice of participants, which grew stronger as the music therapy process developed. This growth in resilience was evident in participants’ remarking that they were becoming open to change and moving forward, accepting their circumstances and experiencing increased motivation for goal setting. They referred to their strength and courage, intention to persist forward, make decisions and willingness to venture, and they were progressively attributing positive qualities to themselves. During the post-therapy interviews, patients reported having experienced music therapy as up-building, fulfilling and strengthening, promoting self-growth and increasing their self-confidence.

### The voice of liberation

‘Take a deep breath and then taking the breath out and then exercising my neck and my shoulders and my arms, my legs, and then I can see my legs – they still can move; my arms, I can move them; I feel free in my body and my heart was not beating like before.’ (Participant J, female, post interview)

‘Yes, I feel liberated, feel that I can do anything.’ (Participant A, female, session 04)

‘I feel alive.’ (Participant D, female, session 01)

‘It energises me.’ (Participant P, female, session 08)

The voice of liberation was primarily sounded through active musical participation. The movement towards liberation was experienced through extending and varying the range of musical expression, a shift from low to higher affective and musical energy states and articulations of music calming stress and tension. Liberation was voiced through spontaneous musical initiative, interactive musical thrill and mutuality, increasing exploration in music-making, increased self-confidence in musical expression, the development of musical material based on patients’ recall of musical material from previous sessions and the creative elaboration of musical qualities. Patients reported during the post-therapy interviews experiences of joy and laughter, peaceful comfort, feeling better, expressing frustration, being rid of anger and irritation and allowing themselves to be free.

## Discussion

The deliberate qualitative approach of this study gave a voice to the subjective experiences of people who may be compromised in having a (clear) voice. Patients revealed (both verbally and musically) 11 salient voices that proclaim struggle, disturbance, feelings, isolation, powerlessness, desires, flow and connection, reflection, symbolism, resilience and liberation.

One may reasonably suspect that these voices also pertained to the mental health users affected by the Life Esidimeni events, but the extent to which this was the case will remain an unanswered question. Insofar as these were in fact their voices too, one may empathically imagine that these events added insult to injury. This underscores the gravity of the finding by the Ombud, that is, a failure to listen.^1^ Averting such failure in future should crucially involve processes instituted at a policy level that ensure listening to the various stakeholders as recommended elsewhere.^18^ True to a person-centred approach,^19^ potentially affected mental healthcare users must be regarded as the principal stakeholders who must be heard in processes to this end.^20^

Listening to mental healthcare users is likely to require deliberate effort. This is also suggested in our study, in which it was not always easy for the patients to speak, but at no stage did they not speak in the broader sense. Emotional, physiological, cognitive and psychological factors may play a role in limiting verbal and musical expression, but despite these, patients gave expression to their experiences.^21,22^

A special kind of listening is required to hear the voices of patients who have difficulty in expressing themselves. It is difficult to really listen to a voice that is, *inter alia*, limited in its capacity to express, disorganised, rigid, devoid of emotion and unable to articulate what lies within. If solely reliant on verbal information, we may not easily hear what the patient has to say. When we extend the patient’s voice beyond the verbal to the musical, the creative and the non-verbal and listen in a more nuanced manner to the ‘what’ and the ‘how’ of the voice, and when we listen to how the patient makes sense of their experience, perhaps then listening would lead to attending to and really hearing what patients have to say and need us to hear.

Lipari^23^ suggests that in listening we become a ‘listening being’, and to truly hear the other, such a stance should be adopted. She articulates this as follows:

Listening is thus a dwelling place from where we offer our ethical response, our hospitality, to the other and the world. Listening being is thus an invitation – a hosting. This hosting of other is as a guest, as a not-me. I don’t have to understand, although you may feel understood. I don’t have to translate your words into familiar categories or ideas. I don’t have to feel what you feel, or know what it feels like to be you. What I do need to do is stand in proximity to your pain. To stand with you, right next to you, and to belong to you, fully present to the ongoing expression of you. Letting go of my ideas about who you are, who I am, what should be. I let all that go, and stay present, attending, and aware. Not indifferent, but in a state of letting go of conceptual thought, what Levinas calls beyond dialogue, and what Heidegger calls releasement.^23^

Listening in this way affords the experience of another finding their voice, of coming into their own through being heard by another. This mode of listening opens up the listening act to possibilities of hearing that which may not normally be heard or otherwise expressed.

Although music therapy provides the means for listening in ways that extend the verbal to a musical domain, the voices identified in this study have also emerged by other means, including ordinary interpersonal communication and other kinds of therapy. Struggle, disturbance, isolation, powerlessness and desire have been well recognised.^24,25^

Regarding the voices of symbolism, desire and flow, this study demonstrates that music therapy evoked symbolic material used in both concrete and abstract ways, with which patients gave voice to their experiences. Symbolic material consistently opened the doorway to personal narrative and association. Unkefer and Thaut^26^ state that no matter how symbolic meaning is derived, it gives music communicative potential in a therapeutic setting. Engagement with symbolic expression gives rise to the individual meaning, which may enable the patient to transcend suffering or gain a new perspective. Symbols deal with universal problems such as life, love and suffering.^27^ The voice in symbols is connected with the voice of flow, expressing regularity and irregularity, interruption and flow, instability and stability, (dis)synchronisation, (loss of) affective resonance, despair and desire, struggle in and from being trapped, impasse, rigidity and losing the capacity for modulation as apt descriptors of the complexities in the lived experiences of patients.^28^

The voices of resilience and liberation are congruent with the resource-oriented strengths-based approach of positive psychiatry.^29,30^ In music therapy, this approach upholds the principles of (1) nurturing strengths, resources and potentials, (2) collaboration rather than a clinician-driven intervention, (3) viewing the individual within their context and (4) taking and using music as a health resource.^31^ The empowering philosophy of this approach focuses on:

[*A*]mplifying strengths rather than mending weaknesses of patients, recognizing competencies related to their therapeutic process of change, nurturing and developing resources of patients through musical interactions and collaborations in the therapeutic process and focusing on musical resources and music as a resource.^32^

Throughout the music therapy process, patients were invited to access inner resources creatively, take initiative, exercise choice, assume leading roles and express thoughts and feelings openly.

### Limitations and evaluation of the findings

The findings here neither purport to represent a conclusive nor an exhaustive voice of patients with MDD and SSD – that would go against the listening stance advocated here and the affordances of qualitative research methods. Whilst extensive qualitative data were obtained across 131 sessions and 15 post-therapy interviews, allowing for in-depth analysis to the point of data saturation, a larger sample may have revealed other or additional emerging themes or voices, even though a sample size of 20, by qualitative standards, is generally considered reasonable for yielding relevant and trustworthy content for the particular setting.

Generalising these findings will depend on the extent to which another context would be similar to this study. This context-specific nature of findings in qualitative research means that findings may be different elsewhere. One contextual specificity was that our study focused intentionally on the public mental health sector, which may be different from the private sector considering the disparities pertaining to the South African context.

Bound by an interpretive epistemology, the findings of the study are presented as an interpreted construction of knowledge to which participants and the researchers contributed conjointly.^7,8^ The participants’ contribution is evident in their agency expressed in terms of voices. Participants were the agents of the voices. More specifically, participants were the agents in struggling, feeling, desiring, reflecting and symbolising. The general contribution and influence on these emerging voices by the researchers were described elsewhere.^6^ More specifically, the space in which these voices emerged had been created by the researchers’ engagement in therapeutic and research processes. Thereby, the space was thus contextually set up in ways in which one may reasonably expect feelings, struggling and disturbance to emerge and be recognised and interpreted conjointly. The joint expectation of improvement further set up the space in which the voices of resilience and liberty emerged and could be so recognised and interpreted conjointly.

The findings of this study were extensively evaluated by applying the EPICURE criteria of Stige et al.,^33^ summarised here.^4^ The researchers *engaged* thoroughly with contextual stakeholders in negotiating logistics and setup at the identified hospital site, as well as ongoing discussions with the referring clinical team and hospital ward during data collection. Extensive data were collected and *processed* systematically, in tandem with a therapeutic process at one site with all sessions and interviews being recorded, rigorously transcribed and analysed, guided by the principle that the analysis should account closely for context and subjective content. The analytic *interpretation* was constrained by a deliberate and pervasive effort to bracket personal, theoretical and clinical knowledge, employ triangulation in maintaining rigour and champion patient subjectivity. Interpretations yielded accordingly were nonetheless accountably refracted through the interdisciplinary lenses of psychiatry and music therapy. In *critique*, this study is the first of its kind and the most extensive qualitative music therapy study in South Africa to date. The rich qualitative themes of this study are subject to the limitations highlighted here. The findings may be *useful* for clinicians to listen better by priming their awareness for recognising similar voices of patients. The music therapy intervention and the ways in which it was researched are clearly described, allowing for replication and adaptation in other contexts and populations. The interdisciplinary nature of this research renders the study *relevant* in contributing to knowledge in the fields of music therapy and psychiatry, suggesting the inclusion of music therapy in multidisciplinary treatment programmes in mental health settings as a complementary and augmentative treatment within standard care. Due protocol was followed in gaining *ethical* approval, obtaining documented participant informed consent and managing the transcription and presentation of data in ways that conceal the identity of the participant.

## Conclusion

This study puts at the centre the voices of patients emerging during and after a course of music therapy in both verbal and musical expressions. The importance of listening to these voices for the therapeutic sake of psychiatric patients stands in stark contrast to the failure to listen as reported in the Ombud’s report on the Life Esidimeni tragedy. In high regard for the humanity and dignity of psychiatric patients as bestowed in the Constitution of South Africa on all its citizens, space should be made for these voices in pursing patients’ co-produced and strengths-based recovery.^34^
